# Emerging Connections between Nuclear Pore Complex Homeostasis and ALS

**DOI:** 10.3390/ijms23031329

**Published:** 2022-01-25

**Authors:** Sunandini Chandra, C. Patrick Lusk

**Affiliations:** Department of Cell Biology, Yale School of Medicine, 295 Congress Ave, New Haven, CT 06520, USA; sunandini.chandra@yale.edu

**Keywords:** *C9ORF72* ALS, NPC injury, CHMP7, ESCRT, nuclear transport, POM121, nuclear quality control

## Abstract

Developing effective treatments for neurodegenerative diseases such as amyotrophic lateral sclerosis (ALS) requires understanding of the underlying pathomechanisms that contribute to the motor neuron loss that defines the disease. As it causes the largest fraction of familial ALS cases, considerable effort has focused on hexanucleotide repeat expansions in the *C9ORF72* gene, which encode toxic repeat RNA and dipeptide repeat (DPR) proteins. Both the repeat RNA and DPRs interact with and perturb multiple elements of the nuclear transport machinery, including shuttling nuclear transport receptors, the Ran GTPase and the nucleoporin proteins (nups) that build the nuclear pore complex (NPC). Here, we consider recent work that describes changes to the molecular composition of the NPC in *C9ORF72* model and patient neurons in the context of quality control mechanisms that function at the nuclear envelope (NE). For example, changes to NPC structure may be caused by the dysregulation of a conserved NE surveillance pathway mediated by the endosomal sorting complexes required for the transport protein, CHMP7. Thus, these studies are introducing NE and NPC quality control pathways as key elements in a pathological cascade that leads to *C9ORF72* ALS, opening entirely new experimental avenues and possibilities for targeted therapeutic intervention.

## 1. Introduction to Amyotrophic Lateral Sclerosis: Background of Genotypic and Pathological Features

Neurodegenerative diseases constitute a spectrum of pathologies that includes amyotrophic lateral sclerosis (ALS), frontotemporal dementia (FTD), Parkinson’s disease, Alzheimer’s disease and Huntington’s disease, which affect millions of people worldwide [1]. The most common motor neuron disease is ALS, also known as Lou Gherig’s disease in honor of the famous New York Yankees baseball player who succumbed to it. ALS is characterized by a loss of motor neurons in the brain and spinal cord, which leads to progressive muscle weakness and atrophy, dysarthria, dysphagia and spasticity [2]. Although there are only two FDA-approved drugs that can slow the progression of symptoms, like all neurodegenerative diseases, there is no cure [3]. Thus, there remains a critical need to understand the underlying pathomechanisms at the molecular level in order to inform new therapeutic strategies.

Efforts to define such mechanisms benefit from genetic studies that, beginning with *SOD1* 28 years ago, have identified several causative genetic variants in a multitude of genes, including *C9ORF72*, *TARDBP*, *FUS*, *ANG*, *MATR3*, *OPTN*, *TBK1*, *NEK1*, *C21ORF2*, *CHCHD10*, *DCTN1*, *TUBA4A*, *PFN1*, *SQSTM1*, *VCP* and *UBQLN2* [4,5]. These genes were identified because of their autosomal dominant pattern of inheritance within families. These “familial” ALS (fALS), however, only constitute ~10% of total ALS cases; the vast majority of ALS cases are sporadic (sALS), even if they sometimes share a known genetic association with fALS [6]. For example, the most common genetic cause of ALS, responsible for 40% of fALS and 8% sALS, is a G_4_C_2_ hexanucleotide repeat expansion (HRE) in the first intron of the *C9ORF72* gene (hereafter referred to as C9-ALS) [7]. This repeat expansion is also common in FTD, the second leading cause of dementia after Alzheimer’s disease, which shares clinical and pathological features with ALS [8]. Thus, because of its central importance to ALS/FTD, there has been considerable interest in defining the underlying mechanisms regarding how the expression of the HREs impact cellular physiology [9].

It is now known that G_4_C_2_ repeats are bidirectionally transcribed to form sense and anti-sense repeat RNA that can then undergo repeat-associated non-ATG translation (RANT) into five dipeptide repeat (DPR) proteins, namely poly(GA), poly(GP), poly(GR), poly(PA) and poly(PR). Both repeat RNAs and DPRs are thought to drive neurotoxicity [10,11]. In fact, the expression of repeat RNAs and DPRs can inhibit cell growth in model organisms such as flies and yeast, hinting that they may impact shared molecular processes between all cells. Indeed, a confluence of evidence over the past few years has strongly implicated nuclear transport machinery as a key target of both repeat RNAs and DPRs (reviewed extensively in [12,13,14,15,16]). Most recently, even the nuclear pore complex (NPC) itself appears to be susceptible to damage caused by expression of HRE repeat RNA [7]. These and other data are raising questions as to the underlying mechanisms that could lead to the removal and degradation of NPC components (nucleoporins or nups) and whether nuclear envelope (NE)-specific quality control mechanisms may be at play. Here, we examine the latest research implicating ALS-specific pathomechanisms that intersect with the nuclear transport machinery in the context of emerging quality control mechanisms that function at the NE. These pathways could provide a roadmap forward to identifying the underlying causes of these diseases.

## 2. Nuclear Transport and NE Quality Control in ALS

### 2.1. The Soluble Nuclear Transport Machinery Is Impacted in ALS

**Figure 1:**
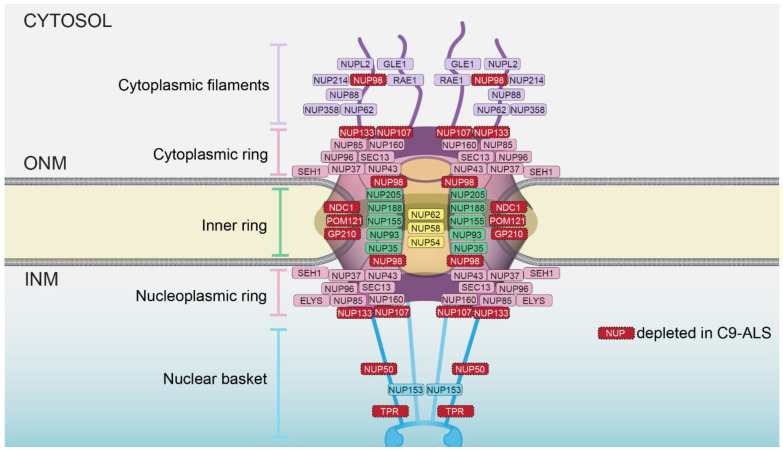
Schematic of NPC embedded in the nuclear envelope. Major architectural assemblies and relative position of individual nups are indicated. Nups, in red, are depleted in C9-ALS. ONM is outer nuclear membrane; INM is inner nuclear membrane.

NPCs are 100 MD protein assemblies that form selective transport channels that span the double membraned NE; there are hundreds to thousands of NPCs that gate the nucleus in a typical mammalian cell, including neurons [17,18,19]. Each NPC is built from ~30 nups that are assembled into subcomplexes that form modular units repeated in 8-fold, radially symmetric, concentric ring assemblies to construct a stable core scaffold architecture (Figure 1). The cytoplasmic ring and nucleoplasmic ring are largely compositionally similar, with notable differences that allow for the anchorage of cytoplasmic filaments/an mRNA export platform on the cytoplasmic side of the NPC and a nuclear basket structure that extends into the nucleus [20]. The scaffold also provides anchor points for intrinsically disordered proteins, rich in FG amino acid residues found in repetitive motifs (the FG-nups) that fill the central transport channel. There are thousands of FG-repeats in the central channel that are principally responsible for nucleocytoplasmic compartmentalization through two core mechanisms: they establish a diffusion barrier that impedes the passage of macromolecules, while also providing binding sites for shuttling nuclear transport receptors (NTR; also known as karyopherins/importins/exportins) that carry signal-bearing cargo through the NPC. Directionality and energy for multiple rounds of transport are provided by the Ran GTPase, which itself is predominantly localized to the nucleus at steady state, bound to GTP [21,22].

There is now an abundance of evidence demonstrating that essentially all elements of nuclear transport machinery, including NTRs, nups and Ran, are mislocalized in either nucleoplasmic or cytosolic aggregates in ALS [12,14,15,23,24]. The biogenesis of these aggregates is not always understood. Some, for example, are stress granules, which can be formed by the DPRs themselves [25,26]. Others might reflect a protective response as the direct binding of NTRs to the DPRs can suppress their pathological interactions with RNA-binding proteins such as TDP-43. Such a mechanism might also be related to the ability of NTRs to promote the disaggregation of pathological FUS and TDP-43 aggregates [27]. This latter role speaks to a fundamental role of NTRs that can act as chaperones that shield binding interfaces that contribute to both productive and pathological phase separation [28]. Indeed, the relationship between the phase-separation behavior of pathological RNA binding proteins such as FUS and TDP-43 (which also bind to and impact the phase separation properties of nups as well [29]) and NTRs is just beginning to be unraveled [28].

### 2.2. NPC Injury in C9-ALS

Although the presence of nuclear and cytosolic aggregates containing nuclear transport components has been well documented, whether the NPCs themselves were altered in C9-ALS is just coming into focus. In fact, such an idea may not have been prioritized as it is well established that the scaffold of the NPC is extremely long- lived in neurons, suggesting that, once assembled, it is difficult to dislodge a nup from the NPC [18,30,31]. Recent work is challenging this idea. Using an induced pluripotent stem-cell-derived neuron (iPSN) model of C9-ALS, Coyne et al. (2020) purified nuclei and examined the localization of 23 nups using immunofluorescence structured illumination microscopy, which can, in principle, resolve individual NPCs [32,33]. Remarkably, 8 of the 23 nups were found at lower levels in nuclear and NE pools (Figure 1). These nup losses were caused by the expression of the HRE RNA, as they were not observed in *C9ORF72* null iPSNs, nor in the context of DPRs. Moreover, both sense (SOs) and antisense oligos (ASOs) that target the RNAs prevented the nup loss [7]. Curiously, these nup depletions spanned all major architectural units of the NPC, including the nuclear basket (NUP50 and TPR), the central transport channel (NUP98), the cytoplasmic/nucleoplasmic rings (NUP107 and NUP133) and all three of the pore membrane proteins (GP210/NUP210, NDC1 and POM121) (Figure 1).

**Figure 2:**
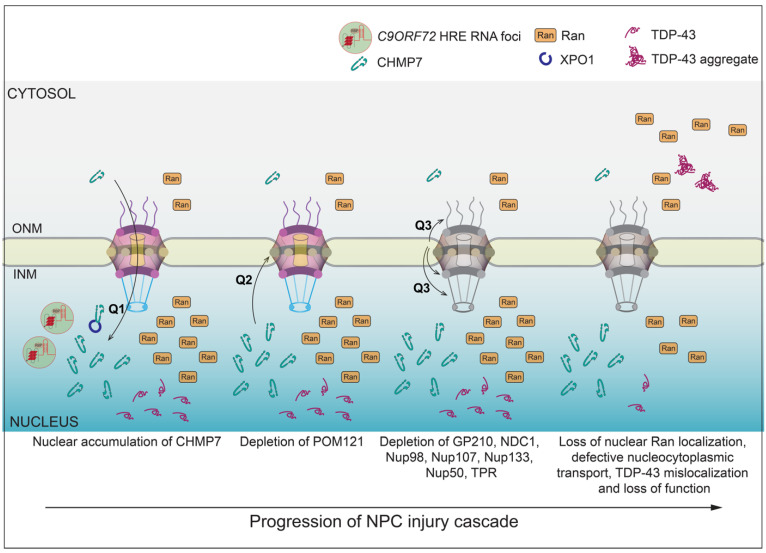
Model of the NPC injury cascade observed in C9-ALS IPSNs with key unknown questions (Q). NPC injury is thought to proceed in a stepwise process (left to right) beginning with an insult that leads to CHMP7 nuclear import or inhibition of its nuclear export by XPO1 (Q1). This aberrant accumulation of CHMP7 leads to the loss of the linchpin POM121 through a mechanism that remains ill defined (Q2), which in turn results in the loss of additional nups (Q3). The overall nup loss burden (depicted as graying of the NPC) is suggested to impact nuclear transport and disrupt Ran and TDP-43 localization.

Consistent with the relevance of these reductions in nup levels to the disease, similar phenotypes were observed in postmortem *C9ORF72* patient motor cortex and spinal cord tissue samples [7]. Perhaps most remarkably, the reduction in nups was not restricted to the familial C9-ALS, but also extended to iPSNs derived from patients with sALS as well [34]. The latter is strongly suggestive that the reduction in specific nups from the NPC may be a foundational pathognomonic feature of ALS more generally. This selective loss of nups was accompanied by a mislocalization of the Ran GTPase, which resulted in dysfunctional active transport of reporter proteins—a common theme in several ALS/FTD studies [13,14,15,16,23,24,35]. This led to a decreased stress-induced neuronal cell viability, along with the aberrant, cytoplasmic accumulation of TDP-43; the latter phenotype being an established pathogenic marker in ALS/FTD. Thus, there is a cascade of events that lead to NPC injury and downstream consequences in the context of *C9ORF72* HRE expression (Figure 2).

### 2.3. How Are Nups Selectively Removed from the NPC?

To answer this question, it is worth considering that the observed loss of nups across multiple subcomplexes is unusual as it runs counter to what is typical, at least for the experimental depletion of nups. In these cases, it is most common for a nup to be co-depleted with its binding partners. Indeed, a drug-inducible, degron-mediated depletion strategy of specific nups revealed that, not only can they be removed from NPCs, but the degradation of a single component of a given subcomplex resulted in the co-depletion of its binding partners. This led to the degradation of whole ring complexes while leaving the other ring complexes intact [36,37,38]. That the observed reduction in a subset of nups does not result in the co-depletion of their subcomplex partners in the C9-ALS scenario is interesting, and one can consider several possibilities to explain this result. The most obvious is that the experimental, degron-mediated degradation of nups is not an effective proxy for C9-ALS-mediated nup loss and unique, yet to be discovered mechanisms are involved. For example, it is possible that the removal of just a few copies of a given nup from the NPC does not lead to a chain reaction that triggers the complete disassembly of an entire ring assembly. Such an idea is in line with scanning-EM data of C9-ALS NPCs [7], which suggest no gross morphological changes to the NPC structure (with the caveat that this approach does not have sufficient resolution to observe subtle changes in NPC structure). A better understanding of morphological and structural alterations of the NPC would certainly benefit from future studies involving in situ cryo-focused-ion-beam milling and electron tomography of ALS nuclei.

An alternative possibility to explain the unique pattern of nup depletion in C9-ALS neurons is that there are, in fact, physical connections between these eight nups (Figure 1) that are yet to be defined. Such an idea is supported by genetic evidence where the re-introduction of overexpressed POM121, and only POM121, is sufficient to restore all eight nups into the NPC in the C9-ALS iPSNs [7]. These data clearly implicate POM121 as a linchpin and might predict physical interactions between POM121 and the eight nups that span multiple subcomplexes (Figure 2, Q3). Consistent with this, POM121 has been shown to physically interact mutually exclusively with nups in both the outer-ring (NUP107–160) and inner-ring (NUP93–205) subcomplexes [39,40]. However, a comprehensive understanding of the POM121 interactome is still needed as, being a membrane protein, it is refractory to biochemical purification and reconstitution experiments that have been possible for most other nups. Such an effort is essential, however, to fully understand why the loss of POM121 is part of the C9-ALS pathomechanism. These efforts must also focus on the human protein, as nup losses are not recapitulated in mouse models where the POM121 is considerably diverged at the DNA sequence level [7].

Regardless of the nups that interact with POM121, there remains a critical missing molecular link between the repeat RNAs and the triggering of the nup loss cascade (Figure 2, Q1). Further, the ultimate mechanism of POM121 degradation remains unknown (Figure 2, Q2). We consider the latter problem first, which informs the former. The clearance of a membrane protein such as POM121 almost certainly utilizes either an ER-associated degradation (ERAD) or autophagy mechanism [41,42,43]. However, in general, neurodegenerative diseases are most often associated with defects in these proteostasis pathways, which leads to the stabilization (not the degradation) of proteins [24,44,45]. In fact, many of the genetic mutations associated with ALS include mutations in genes involved in autophagy (*C9ORF72*, *FIG4*, *OPTN* and *TBK1*), or proteasomal degradation (*UBQLN2*), or both (*SQSTM* and *VCP*) [4,5], and the upregulation of autophagy using the mTOR inhibitor rapamycin is being used as a therapeutic intervention for ALS [46]. As the C9-ALS-mediated nup loss appears to be an aberrant degradation event, this suggests a unique mechanism that might better reflect the dysregulation, or gain of function, of a proteostasis pathway. For further insight into what these pathways might be, we turn to work in non-neuronal model systems, which is revealing that there may be NE-specific factors that contribute to the ERAD and autophagy of NE components such as NPCs and integral inner nuclear membrane (INM) proteins.

### 2.4. NE-Specific Quality Control Mechanisms

In the case of INM-associated degradation (INMAD), there are dedicated E3 ubiquitin ligases that have been discovered in budding yeast that are specific for ubiquitylating integral INM proteins [47,48,49,50]. Whether these ligases are conserved in humans remains to be determined, although there is evidence suggesting that some integral INM proteins are targeted by ERAD mechanisms that release unfolded membrane proteins into the nucleus in human cells, suggesting that INM-specific ERAD machineries are about to be discovered [51]. Further, there are proteasomes attached to the INM in algae [52], yeast [53,54,55,56] and mammalian cells [57]. Yeast systems have also been instrumental in identifying nuclear autophagy pathways that require outer nuclear membrane cargo adaptors that remodel the nuclear membranes and capture INM proteins [58,59] within intralumenal vesicles [58]. The latter mechanism provides a satisfying solution for how INM can be captured by cytosolic autophagy machinery without a loss of nuclear integrity. Likewise, mechanisms of NPC-phagy have been uncovered that also require elaborate membrane remodeling to remove entire NPCs [60,61,62]. Again, whether such mechanisms have any role in the context of ALS remains ill defined, but compelling data implicate that whole NPC removal mechanisms, albeit outside of core autophagy factors, are likely to function in mammalian cells as well [63].

### 2.5. CHMP7 as a Key Player in NPC Injury in ALS

If there is a common molecular thread between NPC removal mechanisms in yeast and in human cells, it is the involvement of endosomal sorting complexes required for transport (ESCRT). ESCRT proteins, in particular a class of ESCRT proteins called ESCRT-III, form spiraling polymers that remodel many organelle membranes away from the cytosol (or nucleoplasm) and drive membrane scission [64]. The ESCRT pathway has been implicated in the removal and sealing-off of defective NPCs in budding yeast [60,65,66,67] and in the turnover of NPCs in mammalian systems as well [63]. It was these connections that prompted an investigation into whether ESCRT proteins, specifically an NE-specific ESCRT called CHMP7, might play a role in nup degradation in C9-ALS [34]. Such an investigation was further bolstered by evidence that several mutations in a core ESCRT-III component gene, *CHMP2B*, have been discovered in ALS/FTD patient tissue samples [68,69,70,71,72,73]. Further, transcriptomic profiling of motor neurons in mice models of spinal and bulbar muscular atrophy (SBMA—another degenerative motor neuron disease) identified that the *CHMP7* transcript was downregulated, potentially implicating CHMP7 function in the disease pathogenesis [74].

CHMP7 is a principal component of an NE surveillance mechanism that monitors the function of NPCs and the integrity of NE membranes [75]. The surveillance system is established by preventing the nuclear accumulation of CHMP7. Indeed, although CHMP7 can passively diffuse through NPCs to enter the nucleus, it is actively exported by the NTR CRM1/XPO1. This export is necessary to prevent CHMP7’s untimely binding and activation by an integral INM protein, LEM2 [67,76]. Thus, in the context of robust nucleocytoplasmic compartmentalization, the surveillance system is found in a poised or primed state, with CHMP7 and LEM2 physically segregated on either side of the NE. In scenarios in which NPCs are defective or there are ruptures to the nuclear membranes, the resulting loss of nucleocytoplasmic compartmentalization leads to the binding of CHMP7 to LEM2, which activates its polymerization [67,77] and membrane remodeling abilities that help reseal the NE [75]. The first clue that this pathway might be compromised in C9-ALS was the observation that CHMP7 is found localized in the nucleus of C9-ALS iPSNs [34] (Figure 2, Q1); more recent evidence indicates that another ESCRT regulatory factor VPS4 is also in the nucleus [78]. Counterintuitively, however, this change in localization occurred before, not after, any detectable nup loss, raising the possibility that CHMP7 was acting as a dominant negative and the aberrant triggering of this surveillance pathway was an input to nup degradation [34] (Figure 2).

The idea that CHMP7 might act as a dominant negative already has precedent in other model systems [66,67,76,79]. For example, by simply preventing its nuclear export by chemically inhibiting XPO1/CRM1, CHMP7 inappropriately localizes to the nucleus, where its binding and activation by LEM2 drives the formation of a fenestrated network of proliferated INM in both yeast [67] and mammalian systems, which may even directly cause DNA damage [76]. Therefore, to test if the aberrant nuclear accumulation of CHMP7 was upstream, and perhaps causative of NPC injury, Coyne et al. overexpressed CHMP7 with mutations in its nuclear export signal (NES) in otherwise normal neurons [34]. Strikingly, the resulting nuclear accumulation of CHMP7 mimicked the pathogenic state as it led to NPC injury and a specific reduction in the eight nups (Figure 1 and Figure 2). In contrast to other systems, however, there was no obvious accumulation of the CHMP7-NES mutant at the INM. Furthermore, consistent with these data, a reduction in LEM2 levels did not lead to NPC injury [34]. The latter results are suggestive of a potentially novel mode of CHMP7 activation, which is likely an early step in the NPC injury cascade (Figure 2). Indeed, using ASOs against CHMP7 in IPSNs derived from both fALS and sALS patients mitigated all aspects of NPC injury and its downstream impact on the Ran GTPase and nuclear transport. Of additional significance, the CHMP7 ASOs also rescued TDP-43 mislocalization (Figure 2). As TDP-43 mislocalization is observed in 97% of all ALS cases, in 50% of FTD cases and in other neurodegenerative diseases [80], it will be important to understand how frequently CHMP7 dysfunction is tied to TDP-43 mislocalization.

### 2.6. What Are the Mechanisms That Trigger CHMP7 Nuclear Accumulation and Ensuing NPC Injury?

As the aberrant accumulation of CHMP7 in the nucleus is likely an early step in C9-ALS pathogenesis, it is worth considering potential insults that could lead to this. One likely possibility is that the *C9ORF72* repeat RNAs prevent the nuclear export of CHMP7 by either directly or indirectly interfering with CHMP7’s interaction with XPO1/CRM1 (Figure 2, Q1). Such interference could be specific to a CHMP7–XPO1 interaction, or their targeting of XPO1/CRM1 could result in the global inhibition of XPO1/CRM1-mediated nuclear export, an analogy to how DPRs directly impede cargo loading of some NTRS [81]. Alternatively, repeat RNA might directly or indirectly potentiate the aberrant activation of CHMP7 in the nucleus. Regardless of the ultimate mechanism, simultaneous efforts must also be undertaken to understand how nuclear CHMP7 actually leads to nup degradation (Figure 2, Q2).

It is most plausible that CHMP7 activity is tied in some way to the removal of POM121 from the NPC (Figure 2, Q2). Although one can imagine direct mechanisms, the lack of any detectable NE accumulation of CHMP7 in the context of C9-ALS iPSNs disfavors this possibility [34]. Thus, it may be more likely that aberrant CHMP7 activity in the nucleus sequesters, or otherwise inhibits, factors that stabilize POM121 incorporation into the NPC (Figure 2, Q2). NTRs may be the key players here as, in addition to its FG-repeats that interact with NTRs, POM121 also has an NLS that binds specifically to the NTR Karyopherin β1/Karyopherin α complex. This interaction is thought to be important for POM121’s unique necessity to the interphase, as opposed to the post-mitotic, NPC assembly mechanism [40,82,83]. Thus, perhaps repeat RNA targeting of Karyopherin β1/Karyopherin α could result in less stable POM121 incorporation into NPCs. Such putative destabilization might not be easily detectable by microscopy early in the NPC injury cascade, but may, nonetheless, be sensed by endogenous cellular factors. For example, recent evidence suggests that the LEM2 paralogue, MAN1, might directly assess the compositional integrity of NPCs [84]. That budding yeast MAN1 also binds to CHMP7 [66] suggests a potential link between an NPC integrity sensing mechanism and CHMP7 that might be worth investigating.

Indeed, it is possible that NE protein(s) more generally serve as sensors of NE and NPC function and couple this role with proteastasis pathways. For example, in Hutchinson–Gilford Progeria Syndrome (HGPS), neuronal nuclei express a mutant lamin A protein called progerin. The aggregation of progerin causes clustering of the INM protein SUN2. As SUN2 reaches into the NE lumen, this clustering leads to the sequestration of lumenal chaperones and the triggering of the unfolded protein response [85]. It is possible that this protective pathway is abrogated as part of an ALS mechanism. For example, the expression of a C-terminal fragment of TDP-43 (TDP-CTF—a major component of cytoplasmic TDP-43 aggregates in ALS/FTD patient brain tissue [86,87]) resulted in the mislocalization of SUN2 [88]. Thus, collectively, these data support that the INM may be a fertile area to investigate ALS mechanisms.

## 3. Conclusions

In closing, defects in the nuclear transport machinery including the loss of nups within the NPC itself are central to both C9-ALS, and likely some sALS, pathogenesis. That CHMP7 has emerged as a potential driver of nup loss (Figure 2) suggests looking for the upstream events that trigger the pathogenic cascade should be a priority for future work. Likewise, ultimately understanding how nup loss leads to neuron dysfunction must also be considered. Historically, the deletion of a small subset of nups without compromising overall NPC structure would not have been predicted to have a profound impact on nuclear transport. This idea, however, rests on the conceptualization of the NPC as a static entity that is refractory to small perturbations, which is now being challenged by a plethora of recent NPC structures that capture NPCs in their native cellular state in algae [89], yeasts [62,90,91] as well as mammalian cells [38,90,92,93]. These structures present compelling evidence that the NPC scaffold is capable of dilating and constricting in response to NE tension [38] and/or the overall energy state of a cell [90]. As such changes would require massive rearrangements within the NPC scaffold, it is easy to imagine how a loss of just a subset of nups could lead to a “jamming” of these kinds of dynamics. Thus, an exciting future for exploring how NPC dysfunction could contribute to ALS pathology would be to more directly explore the impact of nup loss on such NPC dynamics. This work might also inform the function of the NPC dynamics themselves, as it is not yet understood how these structural changes impact the diffusion barrier and active transport properties of the FG-nup collective.
